# Transthoracic echocardiography monitoring during ASD closure using an artificial hand system

**DOI:** 10.1186/s12947-020-00202-5

**Published:** 2020-06-17

**Authors:** Yun-Ching Fu, Shen Kou Tsai, Wen-Yen Jian, Tsung-Cheng Shyu, Chieh-Mao Chuang, Betau Hwang

**Affiliations:** 1 Pediatric Cardiology, Department of Pediatrics, Children’s Hospital, China Medical University, No. 2, Yude Road, North District, Taichung, Taiwan, ROC; 2 Cheng-Hsin General Hospital and National Taiwan University, No 45, Cheng Hsin St., Beitou, Taipei, Taiwan, ROC; 3 Pediatric Cardiology, Heart Center, Cheng-Hsin General Hospital, No 45, Cheng Hsin St., Beitou, Taipei, Taiwan (ROC); 4 Pediatric Cardiac Medical Center, Tung’s Taichung MetroHarbor Hospital, No 45, Cheng Hsin St., Beitou, Taichung, Taiwan (ROC)

**Keywords:** Artificial hand, Real-time transthoracic echocardiography monitoring, Transcatheter ASD closure, Transesophageal echocardiography, Intracardiac echocardiography

## Abstract

**Aim:**

Continuous real-time echocardiographic monitoring is essential for guidance during ASD closure. However, transthoracic echocardiography (TTE) can only be implemented intermittently during fluoroscopy. We evaluate a novel approach to provide real-time imaging during the entire procedure.

**Finding:**

We developed a custom-made TTE monitoring apparatus using artificial hand (AH-TTE) that enables real-time TTE images during atrial septal defect (ASD) closure. Thirty-two patients underwent successful device implantation using AH-TTE monitoring without complications. The median duration for real-time AH-TTE monitoring was 22 min and the median fluoroscopy time was 7.2 min. One case of pericardial effusion and one of transient bradycardia event due to air embolism was detected. All patients had uneventful recoveries.

**Conclusions:**

Our simple and novel monitoring technique with AH-TTE provides TEE-like monitoring and may be a new alternative method for ASD closure. It gives real-time stable TTE images and minimizes radiation exposure for the interventional team during fluoroscopy.

## Introduction

Transesophageal echocardiography (TEE) has become the mainstay imaging modality to guide structural heart interventions [1–11]. Recently, a minimalist transcatheter approach using transthoracic echocardiography (TTE) guidance has been advocated for transfemoral transcatheter aortic valve replacement (TAVR) [12, 13] and atrial septal defect (ASD) closure [14, 15]. Using TTE has the advantages of a shorter procedural time, hospital stay and lower costs due to the avoidance of general anesthesia. However, TTE only guidance should not be performed during live fluoroscopy and thus can only be implemented intermittently, precluding continuous imaging guidance. We therefore developed an AH-TTE monitoring technique that gives stable continuous TTE images for ASD closing during live fluoroscopy. This study assessed the feasibility and safety of this simple, novel custom-made AH-TTE monitoring system.

## Methods

### Artificial hand--transthoracic echocardiography (AH-TTE) monitoring system

**Fig. 1:**
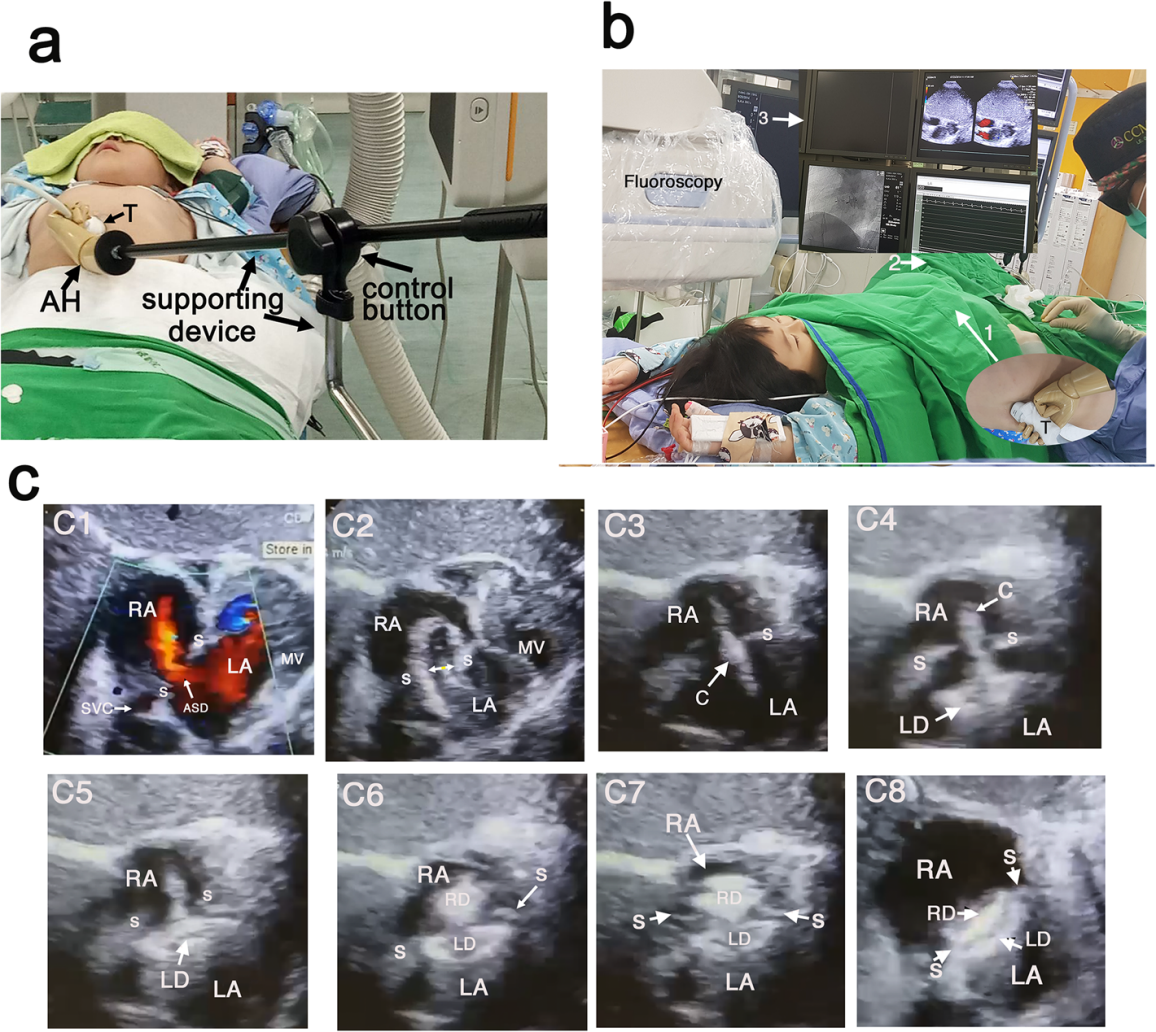
**a** The AH-TTE monitoring system consists of an artificial hand (AH) and a supporting device. The transthoracic transducer is held by the AH and secured in place at the subcostal area by locking the control button to obtain subcostal views and provide real-time TTE imaging during the entire procedure. **b** A 4-year-old girl during ASD closure using AH-TTE monitoring system. The AH grasping the transducer was placed in the subcostal area (arrow 1: AH; arrow 2: supporting device) and the procedure was performed after the area was covered with an aseptic cloth. **c** The correspondent real-time TTE imagings (arrow 3 in B) were demonstrated during ASD closure procedure. C1: An ASD (11.5 mm in size) with a left-to-right shunt. C2: The waist length of the ASD was 11.3 mm (denoted by the two-way arrow) as determined by balloon sizing. C3: The correct passing of the catheter through the defect. C4: The left disc (LD) was deployed in the LA. C5: The LD was pulled back against the atrial septum. C6: The RD was opened in the RA. C7: The RD was deployed in the RA. C8: An 11-mm Amplatzer™ Septal Occluder was successfully implanted and positioned in the atrial septum without requiring shunting. Abbreviations: AH, artificial hand; R, right; L, left; O, occluder; TTE, transthoracic echocardiography; RD, right disc; LD, left disc; RA, right atrium; LA, left atrium; T, transducer; S, septum

The AH-TTE monitoring system consists of an artificial hand (AH) and supportive device (Fig. 1a). AH is made of acrylic with adjustable finger and wrist joints. One end of the artificial arm is connected to the AH and the other end to a manual handle. The AH grasps the transducer, which can be moved by adjusting the wrist to obtain the desired echocardiography views. The manual handle remotely controls the movement of the AH. A zigzag-shaped pole acts as a vertical supporting device, one end of which is affixed to a control knob that securely adjusts the artificial arm’s length and angle, while the other end is affixed to the side rail of the operating table. This prevents any movement of the transducer when placed at the chest wall for the fluoroscopic procedure, despite any movement of the operating table. The transthoracic transducer is held steady by the AH and secured in place at the subcostal region (Fig. 1b) or left chest wall by locking the control knob to obtain a subcostal or four-chamber view providing real-time TTE monitoring.

### Echocardiography protocol

Our AH-TTE monitoring system is suitable for all commercially available transthoracic transducers. We used the ACUSON 4V1c transducer and Philips X5-1, S8-3 and X7-2 (for three-dimensional 3D echocardiography) transducers; the ACUSON transducer was connected to a Siemens healthineers’ ultrasound imaging system, while the Philips transducers were connected to a Philips iE33 or EPIQ ultrasound system (Philips Healthcare, Andover, MA).

**Fig. 2:**
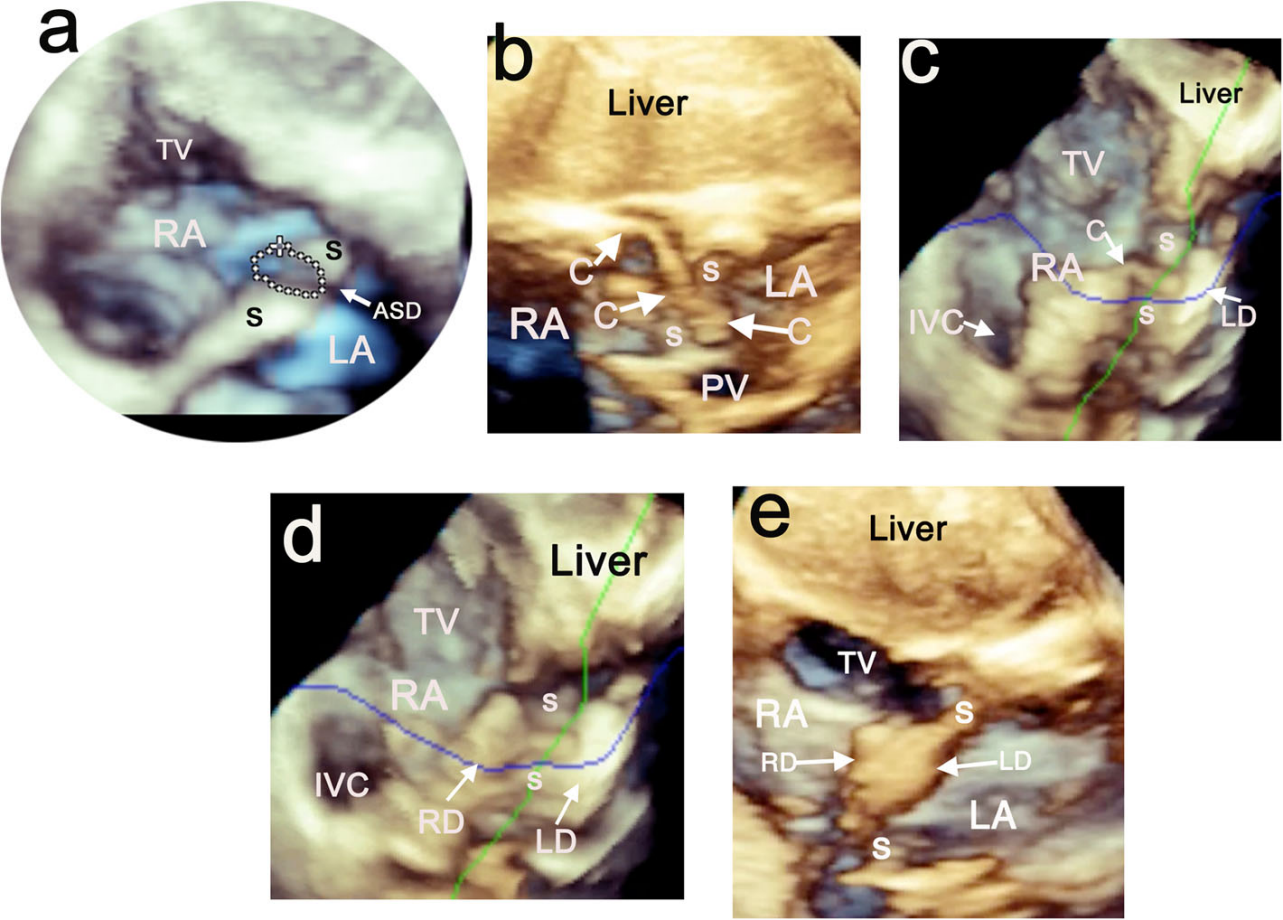
Subcostal views of 3D TTE of MPR images using the AH technique in a 4-year-old boy during ASD closure. **a** The en face oblique atrial view shows a very thick atrial septum (S) and an ovoid-shaped defect measuring 4 × 8 mm in size. **b** The entire route of the delivery cable (C) passing correctly from the RA across the atrial septum to the LA. **c** The LD was deployed in the LA and pushed against the atrial septum (S). **d** The RD was deployed in the RA. **e** The occluder was deployed in the approximate position and its final position was confirmed by TEE images. Abbreviations: AH, artificial hand; TTE, transthoracic echocardiography; MPR, multiplanar reformatting; ASD, atrial septal defect; TV, tricuspid valve; C, delivery cable; PV, pulmonary vein; IVC, inferior vena cava; RD, right disc; LD, left disc

A multiplanar reformatting (MPR) technique was used for the 3D TTE reconstruction using the Philips EPIQ7 QLab ultrasound system (Fig. 2). The subcostal view is the preferred image for delineating the atrial septum (Videos 1, 2 and 3); a modified four-chamber view is also appropriate. Real-time AH-TTE monitoring and fluoroscopy images were displayed simultaneously on multiple integrated screens during the procedure (Fig. 1c).

### Patients

The study protocol was approved by the Institutional Review Board of China Medical University (CMUH107-RECI-170). All patients (or their parents or guardians) gave their written informed consent prior to study participation. A total of 32 consecutive patients with a secundum ASD scheduled to undergo device closure were enrolled into this study. The median age was 7 years (range: 2–60), the median weight was 20 kg (range: 8.6–87), the median ASD size was 5.5 mm (range: 3–21) and the median QP/QS was 1.5 (range 1.1–3.8). All study participants underwent standard monitoring of blood pressure, heart rate and oxygen saturation. Device implantation was performed as previously described [2, 3]. In our study, the implantation procedure was performed under sedation (intravenous 0.1 mg/kg midazolam and ketamine) in spontaneously breathing pediatric patients aged less than 15 years (*n* = 22), as per previously described procedures [16], while the remaining 10 patients were awake with local anesthesia at the puncture site. The same AH transducer self-supporting system was used in all patients.

## Results

All septal occluders were successfully implanted in 32 patients with ASD under the guidance of fluoroscopy and AH- TTE monitoring system. Real-time TTE images using the AH technique provided guidance during the procedure on the size of the defect, crossing of the guided wire, balloon sizing, the delivery of the catheter, deployment of the device, as well as assessments on its position for closure of ASD (Fig. 1c). Images depicting the 3D MPR technique for guiding closure of ASD are shown in Fig. 2. The septal occluder was released only after confirmation from real-time TTE imaging showing that the device was correctly positioned without residual shunting. No complications relating to the AH-TTE monitoring system occurred during the procedure. The median size of the implanted Amplatzer Septal Occluder was 8 mm (range: 4–26). Two 16-mm “Cribriform” septal occluders were used for one patient with multiple ASDs and in another with a fenestrated atrial septum. The median duration for AH-TTE monitoring was 22 min (range: 11–60) and the median fluoroscopy time was 7.2 min (range: 3–23.3). During the procedure, AH-TTE monitoring detected one case of pericardial effusion and one of transient bradycardia event due to an air embolism. No cases of device migration were recorded and all patients had uneventful recoveries in the ward without ICU care. The transducer had to be repositioned in three awaking patients after excessive coughing or movement during the procedure.

## Discussion

During transcatheter ASD closure, TEE [1–8] and Intracardiac echocardiography (ICE) [17, 18] are the routine imaging modalities to guide the procedure. Both can reduce the fluoroscopy time and facilitate the procedure. However, TEE is a semi-invasive procedure requiring general anesthesia as well as endotracheal intubation resulting in increased procedural time and cost. ICE is a new echocardiography modality for guiding ASD closure. ICE eliminates the major disadvantage of the need for general anesthesia related to the use of TEE guidance for ASD closure. But the disadvantages of ICE imaging include the need for a second venous sheath insertion and the risk of inducing transient atrial arrhythmias. The additional costs (2500 US dollars for each ICE catheter) and the need for specific skills are additional limitations [17, 18]. A minimalist approach using TTE guidance has therefore been recommended for transcatheter interventions in TAVR [12, 13] and ASD closure [14, 15] due to the associated advantages of lower costs and superior outcomes for patients. However, TTE only guidance should not be performed during live fluoroscopy and thus can only be implemented intermittently, precluding continuous imaging guidance.

Our results demonstrate that custom-made AH-TTE monitoring system effectively provides continuous real-time TTE images during a minimalist approach of ASD closure. AH-TTE monitoring technique is a simple and non-invasive, yet provides TEE-like monitoring, without the need for general anesthesia during ASD closure. This results in a shorter, more tolerable procedure, without the need for ICU follow-up care. In our study, one patient developed a transient bradycardia event during the implantation procedure and an air embolism was noted on the real-time TTE monitoring, which enabled the clinician to perform emergent aspiration, preventing intraoperative complications. Our AH-TTE monitoring system does not interfere with fluoroscopic images or obstruct fluoroscopic projections during the ASD closure procedure.

Our custom-made AH-TTE monitoring system acts as an extra hand that enables easy application and repositioning of the transducer at any position of the chest wall or subcostal area, saving human effort and associated costs. This technique minimizes radiation exposure for the interventional team (mainly the sonographers); however, the interventionalist would not need additional training in TTE imaging (acquisition and interpretation) to properly utilize this method.

### Limitations

The accuracy of TTE may be compromised when performed in obese patients, especially for adults who tend to have poor acoustic windows that render it difficult to obtain ideal images. The quality of the subcostal view is also influenced by deep breathing, coughing, or moving during the procedure. Therefore, conscious sedation is recommended to prevent this drawback.

## Conclusion

Our novel, simple and non-invasive custom-made AH-TTE monitoring has the potential to be used under live fluoroscopy during ASD closure. It can provide continuous TTE images that facilitate the surgical procedure and detect any complications in a timely fashion. Our real-time TTE imaging using the AH-TTE monitoring technique is similar to those provided by the TEE monitoring for ASD closure, while avoiding the need for general anesthesia. Thus, AH-TTE monitoring can facilitate and safely shorten the duration of implantation time and also to prevent radiation exposure of the interventional team during fluoroscope. Therefore, our custom-made AH-TTE monitoring system is an eminently suitable device for continuous TTE images during minimalist transcatheter therapy procedures and arguably has the potential to become the alternative care.

## Supplementary information

**Additional file 1: Video 1.** The cardiologist initially identified the ASD by TTE in the subcostal view.

**Additional file 2: Video 2.** An echocardiographer assisted with the placement of the AH holding the probe to obtain a satisfactory subcostal view.

**Additional file 3: Video 3.** Continuous, real-time TTE images obtained from the AH transducer self-supporting system guided the operating team throughout the transcatheter ASD closure procedure, enabling close monitoring for any intraoperative complications. Abbreviation: AH, artificial hand

## Data Availability

Not applicable.

## Abbreviations

AH: Artificial hand
ASD: Atrial septal defect
MPR: Multiplanar reformatting
ICE: Intracardiac echocardiography
TEE: Transesophageal echocardiography
TAVR: Transfemoral transcatheter aortic valve replacement
TTE: Transthoracic echocardiography
